# Changes Induced by Mind–Body Intervention Including Epigenetic Marks and Its Effects on Diabetes

**DOI:** 10.3390/ijms22031317

**Published:** 2021-01-28

**Authors:** Hyun-Jeong Yang, Eugene Koh, Min-Kyu Sung, Hojung Kang

**Affiliations:** 1 Korea Institute of Brain Science, Seoul 06022, Korea; mrsay@daum.net (M.-K.S.); kds4998@naver.com (H.K.); 2 Department of Integrative Health Care, University of Brain Education, Cheonan 31228, Korea; 3 Temasek Life Sciences Laboratories, Singapore 117604, Singapore; eugene@tll.org.sg

**Keywords:** mind–body intervention, epigenetic modification, diabetes

## Abstract

Studies have evidenced that epigenetic marks associated with type 2 diabetes (T2D) can be inherited from parents or acquired through fetal and early-life events, as well as through lifelong environments or lifestyles, which can increase the risk of diabetes in adulthood. However, epigenetic modifications are reversible, and can be altered through proper intervention, thus mitigating the risk factors of T2D. Mind–body intervention (MBI) refers to interventions like meditation, yoga, and qigong, which deal with both physical and mental well-being. MBI not only induces psychological changes, such as alleviation of depression, anxiety, and stress, but also physiological changes like parasympathetic activation, lower cortisol secretion, reduced inflammation, and aging rate delay, which are all risk factors for T2D. Notably, MBI has been reported to reduce blood glucose in patients with T2D. Herein, based on recent findings, we review the effects of MBI on diabetes and the mechanisms involved, including epigenetic modifications.

## 1. Introduction

### 1.1. Epigenetics

Epigenetic mechanisms allow control of gene activity without altering the DNA sequence, and through this process, genes are able to adapt to the changing environment [1]. Epigenetic information is either inherited or acquired. They might exert long-term effects but have been shown to be reversible. Any exposure before and during pregnancy can affect the parental germ cells and the fetus, inducing epigenetic changes. Besides these, the environment or lifestyle could also cause epigenetic changes in an individual. Epigenetic marks can be divided into three main types: DNA methylation, histone modification, and small non-coding RNA. These epigenetic modifications are spatially and temporally controlled and exhibit gene-expression regulatory functions. For example, the addition of methyl groups to cytosine can stimulate chromatin condensation, causing the transcriptional machinery to lose access to DNA, thus suppressing gene expression. The environment of subjects, such as exercise, diet, and stress, can increase or decrease the methylation modification in the target genomic region, followed by reduction or increase in the corresponding gene activity, respectively. For example, six months of exercise intervention increased DNA methylation of some genes in human adipose tissue, including several candidate genes related to diabetes, with a notable decrease in the corresponding mRNA expression [2]. In contrast, Barrѐs et al. [3] revealed that one bout of exercise reduces the promoter DNA methylation of substrate metabolite genes in the human skeletal muscle, and increases their gene activity. Similarly, acetylation and deacetylation of histones cause chromatin to become loose or tight, respectively, to activate or inhibit gene transcription along the genome. Moreover, microRNA controls the stability of mRNA and access to the translation machinery, thereby affecting protein production [4].

### 1.2. Epigenetic Changes and Diabetes

Type 2 diabetes (T2D) is characterized by a chronic increase in blood glucose level, which is caused by inadequate insulin secretion or insulin resistance. Aging, a sedentary lifestyle, and obesity are all well-known contributors to insulin resistance. The pancreatic islet cells, which secrete insulin, become dysfunctional in insulin regulation after prolonged exposure to high levels of lipids and glucose [5,6].

#### 1.2.1. Diabetes-Related Epigenetic Changes in Parents, and During Prenatal and Early Life 

Notably, individuals with diabetes have been observed to have significant changes related to DNA methylation in the insulin-producing (pancreatic islets) and insulin-targeted tissues (adipose tissue, skeletal muscle, liver). This finding suggests that the epigenetic mark is associated with the incidence of T2D [7,8]. As follows, studies have revealed that these epigenetic marks can be inherited from parents or acquired during fetal or early life and through lifelong environment or lifestyle.

Epigenetic information can be passed on to the offspring by changing the reproductive cells of the parental generation. The pups of male mice on a high-lipid diet exhibited an altered metabolism phenotype, including obesity and beta cell dysfunction [9]. Moreover, environment-induced parental stress can cause epigenetic changes. A restraint stress mouse model revealed that the increased glucocorticoid level of stressed parent mice caused excessive DNA methylation in the *Sfmbt2* gene promoter in sperm cells, which induced hyperglycemia in the offspring by increasing gluconeogenesis through reduced miR-488b-3p expression, followed by enhanced expression of PEPCK [10]. This finding indicated that epigenetic marks acquired due to parental stress conditions can be passed down to their offspring.

The fetus is vulnerable to epigenetic changes depending on the environment. During the fetal development, individuals exposed to conditions such as malnutrition, xenobiotic expansion, substance use, placental insufficiency, gestational diabetes or prenatal stress have been noted to have abnormalities in glucose and lipid metabolism besides a higher risk of developing T2D [11]. A rat model revealed that a mother’s low-protein diet changed the expression of certain transcription factors in the fetal pancreas, inhibiting beta cell proliferation and promoting cell differentiation [12]. Consequently, the number of beta cells decreased in the offspring, thereby increasing the risk of T2D during adulthood. Similarly, several reports have implied that intrauterine exposure increases the risk of T2D in humans. Children born to mothers with T2D during pregnancy were more likely to develop T2D and obesity than those born to non-diabetic mothers [13,14]. Moreover, people exposed to famine during fetal stage were noted to have glucose intolerance in adulthood [15].

Maternal antenatal stress has also been noted to affect body weight and glucose metabolism in the offspring [16]. According to a meta-analysis, body mass index (BMI) (18 studies) and body fat (5 studies) were significantly higher when under fetal stress [17]. In the placenta, HSD11B2 exists to reduce exposure to the maternal glucocorticoid hormone, converting cortisol or corticosterone into inactive metabolites. However, the maternal stress experienced during the prenatal period can induce an increase in DNA methylation of certain CpG sites located in the *HSD11B2* gene promoter and downregulate expression of the enzyme in the placenta [18]. Notably, both human and animal models have observed epigenetic changes after prenatal stress in fetuses and children [18,19] including methylation changes of the glucocorticoid receptor gene (*NR3C1*, receptor for cortisol). For example, changes in *NR3C1* promoter methylation were detected in the cord blood of newborns born to a mother with depression during pregnancy [20]. Moreover, newborns exposed to prenatal stress were noted to have methylation in the *NR3C1* promoter in umbilical cord blood samples [21]. 

The risk factors of T2D were evidenced to be induced not only by the lifestyle during adulthood, but also by the living conditions during early life [22,23]. The epigenetic mechanism associated with the regulation of gene expression plays a crucial role in mediating the connection between early-life adverse conditions and the risk of chronic diseases (including T2D) occurring in the later years of life [24]. Notably, these effects are not solely limited to physical adversity, but also include mentally harmful environments during development. Early-life adversity, such as childhood abuse, consistently exhibits a condition wherein inflammation develops, because of regulatory dysfunction in the inflammatory pathway over a prolonged period of time [25,26]. Chronic mild inflammation is critically associated with the incidence of T2D [27]. Early-life experiences might significantly affect aging-related phenotypes through the epigenetic factors and potentially influence other aging-related diseases [11].

#### 1.2.2. Psychological Stress and Type 2 Diabetes

##### Psychological Factors Related to Type 2 Diabetes

Psychological stress (including depression, anxiety, and anger) is commonly associated with several physical diseases and has been increasingly recognized as a risk factor for disease onset and progression. Studies have suggested that stress plays a causative role in T2D, serves as a predictor of T2D onset, and acts as a prognostic factor in patients with conventional T2D [28]. This finding could be because glucose homeostasis is affected by the cortisol produced by the hypothalamic–pituitary–adrenal (HPA) axis activation during stress [29]. Moreover, psychological stress can reduce the motivation of individuals to sustain a healthy lifestyle. In a study which followed 7000 healthy adults for 10 years, the perceived stress was related to unhealthy behaviors such as physical inactivity, unsuccessful smoking/alcohol cessation attempts, and T2D incidence [30].

Depression is the most studied psychological factor in the field of diabetes. A meta-analysis of people with diabetes revealed that comorbid depression increased the non-adherence to healthy behaviors related to diet, medication, and exercise [31]. Therefore, the unhealthy effects of depression on these behaviors are likely to be detrimental to people with diabetes. Notably, meta-analysis and prospective cohort studies suggest that depression is associated with an increased risk of diabetes [32,33,34]. In addition, depressive symptoms, including lack of joy, despair, and a diagnosis of clinical depression, are considered predictive factors in the development of diabetes [33,34]. Furthermore, negative personality traits, such as anger, have been studied regarding T2D development [35,36]. A 6-year longitudinal study involving 11,615 non-diabetic adults revealed that anger was associated with a high risk of future T2D development [35]. Furthermore, an 11.4-year study involving 5598 adults (no T2D or cardiovascular disease) revealed that anger and anger response significantly increased the T2D risk [36], indicating that anger is a risk factor for developing diabetes.

Positive psychological factors also seem to affect the glycemic control. A study involving 111 patients with diabetes (both type 1 and 2 diabetes) examined the longitudinal relationship between resilience and glycemic control and noted that low stress resilience further aggravated a 1-year follow-up Hemoglobin A1c (HbA1c, glycated hemoglobin) in both types of diabetes [37]. In a longitudinal study involving 97 elderly women (without diabetes), the relationship between positive well-being and glycemic control was investigated [38]. Those with greater positive well-being at baseline exhibited a statistically lower level of HbA1c at a 2-year follow-up. These results suggest that negative psychological factors, such as depression, anger, and low stress resilience increase the risk of diabetes, whereas positive psychological factors, such as positive well-being, have the opposite effect. Psychological stress causes physiological changes through three major pathways, namely the neuroendocrine (cortisol), autonomic, and inflammatory pathways. Therefore, it seems that psychological stress functions through these pathways when it acts as a risk factor for diabetes [28].

##### Cortisol and Type 2 Diabetes

Corticosterone is a primary glucocorticoid in the physiological stress-response system of rodents [39]. Notably, in rodents, chronic administration of corticosterone induces hyperglycemia, insulin resistance, and dyslipidemia [40,41]. In humans, cortisol, a glucocorticoid hormone, is secreted from the adrenal cortex as an output of the HPA axis during stress. Chronic activation of the HPA axis leads to dysregulated cortisol output [42]. Glucocorticoid receptors are expressed in the pancreatic beta cells that secrete insulin, and thus, cortisol stimulation directly affects insulin sensitivity and reduces insulin secretion [43]. Therefore, abnormal cortisol secretion can cause problems with blood glucose regulation, which is why patients with Cushing’s syndrome, those with chronic excessive cortisol secretion [44], and those taking glucocorticoids prescription [45] are often noted to have a high vulnerability to hyperglycemia and have a higher risk of developing diabetes mellitus. A longitudinal study involving 3270 healthy people observed that high levels of evening cortisol were associated with the likely development of T2D within 9 years [46]. Besides the incidence of T2D, upon considering the prediabetic condition (impaired fasting glucose) into the analysis, elevated evening levels of cortisol and a flatter slope of cortisol across the day were noted to be predictive factors of diabetes. However, morning levels of cortisol and cortisol awakening response were not related to T2D onset [46].

##### Autonomic Nervous System and Type 2 Diabetes

Stress-induced sympathetic activation causes changes in blood pressure, heart rate, and cardiac output, which are recognized risk factors for diabetes [47]. A study involving a cohort of 4.1 million adults who did not have diabetes or cardiovascular disease investigated the link between diabetes risk and blood pressure, using the electronic health record connected to the United Kingdom primary care system, and revealed that systolic and diastolic blood pressures were both risk factors for developing diabetes mellitus [48]. Besides blood pressure, an increased resting heart rate and a decreased heart rate variability were considered to be risk factors for T2D. A meta-analysis that investigated 10 cohort studies (120,000 participants) showed a positive relationship between resting heart rate and incident of T2D [49]. Changes in the autonomic nervous system (increased sympathetic nervous system and decreased parasympathetic nervous system), which increased the risk of T2D, were associated with metabolic syndrome [50], and decreased heart rate variability (markers of autonomic nervous system control) was associated with increased levels of fasting blood glucose (FBG), cortisol, and expression of pro-inflammatory cytokines [51]. 

##### Inflammation and Type 2 Diabetes

Chronic inflammation resulting from abnormal immune system activation is a risk factor for diabetes mellitus. T2D is considered a chronic low-grade inflammatory state associated with multiple inflammatory mechanisms and metabolic pathways [52]. Studies have revealed that circulating concentrations of pro-inflammatory adipokines are increased in patients with T2D. For example, a study involving 15,000 people in Germany reported a dose–response relationship between the impaired glucose status and adipokine concentrations [53]. In addition, a meta-analysis involving 10 prospective studies revealed that an increased concentration of inflammatory cytokines, interleukin (IL)-6, and C-reactive protein (CRP) in the circulatory system was associated with increased risk of future T2D [54]. Indeed, in patients with T2D, the biomarkers indicating chronic inflammation are repeatedly detected in the pancreas, liver, fat tissue, and white blood cells [52].

##### Complications 

Studies suggest that psychological factors, especially depression, increase the risk of complications from T2D. Patients diagnosed with diabetes and depression have higher risk of microvascular [55,56], macrovascular comorbidities [57,58,59], and mortality [60]. Notably, these vascular complications in patients with diabetes appear to be linked to epigenetic changes [61,62,63]. For example, in the genome-wide DNA methylation profiles of DNA isolated from whole blood of myocardial infarction patients or control subjects, two DNA methylation sites were identified to be significantly correlated with myocardial infarction [63]. 

#### 1.2.3. Aging and Type 2 Diabetes

T2D is considered a typical aging-related disease because it generally emerges after the age of 40 years. Because conditions associated with aging processes (e.g., inflammatory states) are characteristics of both T2D and aging [64], T2D is conceptualized as early maturity or accelerated aging [65]. Notably, epigenetic changes are strongly associated with aging. The genome either gains or loses methylation over time. Fraga et al. [66] noted that the epigenome in the cells of young identical twin pairs is similar, whereas the epigenome diverges in the older identical twin pairs, indicating the effect of age on DNA methylation. Moreover, DNA methylation of 3470 sites was revealed to be changed in common across various cell types (fat tissue, liver, and blood) during aging [67]. In addition, in several genes (*FHL2*, *ELOVL2*, *KLF14*) associated with T2D, the methylation of CpG sites were noted to be similarly affected in all investigated tissues.

#### 1.2.4. Lifestyle and Type 2 Diabetes

Over the past few decades, the incidence of T2D has dramatically increased worldwide. Rather than being explained by genetic changes, it is suggested that this was induced by rapid changes in lifestyle globally [68]. According to the study which meta-analyzed nine trials regarding the correlation between total daily sitting time and cardiovascular disease or diabetes in 448,285 participants, it was found that daily sitting time was positively correlated with an increased risk of cardiovascular disease and diabetes [69]. Therefore, a sedentary lifestyle seems to increase the risk of cardiovascular problems and diabetes. Therefore, unhealthy lifestyles, including unhealthy eating, lack of exercise, and smoking, often exacerbate biological changes induced by chronic stress [70].

### 1.3. Types of Mind–Body Intervention and Their Effects

Mind–body intervention (MBI, also known as mind–body training, mind–body practices, and mind–body therapy) refers to meditation, yoga, and tai chi that deal with both physical and mental well-being [71,72]. These interventions are performed with the goal of gaining positive influence on overall health by fostering mental serenity, mental care, and critical cognition, as well as by improving body function through breathing and physical movement. MBI can be categorized into static methods (sitting meditation), dynamic methods (moving meditation), and a combination of both. Static methods can include mindfulness meditation, Vipassana, transcendental meditation (TM), Zen meditation, Buddhist meditation, Sudarshan Kriya, Kirtan Kriya, Pranayama, and relaxation response. Mindfulness meditation is a well-known way to cultivate a state of mindfulness in everyday life [73]. TM is a form of silent mantra meditation with one’s eyes closed [74]. Relaxation response is a simple, secular version of TM [75]. Zen meditation, one of the Buddhist practices, is the practice of sitting cross-legged, concentrating on the mind, and contemplating quietly, and it suspends all judgmental thinking and letting words, ideas, images, and thoughts pass by without getting involved in them [76]. In terms of content, the static method can be divided into open monitoring meditation (e.g., mindfulness meditation) and focused attention meditation (e.g., TM, brain wave vibration).

Dynamic MBIs include movement meditations, such as yoga, tai chi, and qigong, which can be considered a combination of mindfulness intervention and physical activity [77]. Yoga is a group of physical, mental, and spiritual practices or disciplines, largely consisting of different yogic postures [78]. Tai chi is a moving meditation involving a series of slow, gentle motions that are patterned on the movements in nature. Qigong is often referred to as the “internal” portion of tai chi and is characterized by stationary movements that are repeated a certain number of times. 

Combined protocols involve a mix of both static and movement meditations. Mindfulness-based stress reduction (MBSR) is an 8-week integrated training consisting of mindfulness meditation, concentrative meditation, breathing exercises, yoga, autogenic training, and Buddhist philosophy [79]. It blends various techniques and is referred to in the clinical setting as mindful awareness practices [80], mindfulness-based movement [81], mindfulness-based interventions [73], and so on. Buddhist walking meditation is a way of walking with a sense of awakening to one’s body and awareness of the surrounding environment [82]. Brain wave vibration meditation (also known as brain education meditation (BEM)) is a combination of static and dynamic methods that manages health of body and mind based on the following five steps: (1) Brain sensitizing (activating the connection between the body and the brain through various body movements), (2) brain versatilizing (making one’s body flexible through yoga, breathing exercises), (3) brain refreshing (brain wave vibration, energy dance), (4) brain integrating (imagery meditation, body scan), and (5) brain mastering (philosophy of enlightenment) [83,84]. 

MBI has been reported to relieve stress-dependent symptoms of various diseases, including psychological disorders (mood and anxiety disorders), inflammatory diseases, aging, and cancer [80,85,86]. The incidence and progression of diabetes can be affected by stress [46]. Therefore, MBI can be beneficial especially in patients with diabetes. In this work, we explored how MBI affects the incidence and progression of diabetes, as well as exploring its mechanisms, with a special focus on the epigenetic mechanisms.

## 2. Epigenetic Changes Induced by Mind–Body Intervention and Their Effects on Diabetes

### 2.1. Effects of Mind–Body Intervention on Diabetes

Studies that meta-analyzed the effectiveness of MBI on patients with diabetes revealed a consistent efficacy in blood glucose control and lipid metabolism, albeit with some differences in results (Table 1).

#### 2.1.1. Effects of Moving Meditation on Diabetes

Several studies have revealed that moving meditations, such as tai chi, yoga, and qigong are effective in controlling blood glucose in patients with T2D [89,90,94,101]. According to a meta-analysis of 21 trials regarding moving meditation in patients with T2D, moving meditation was noted to significantly improve FBG, HbA1c, postprandial blood glucose (PPBG), total cholesterol (TC), low-density lipoprotein cholesterol (LDL-C), and high-density lipoprotein cholesterol (HDL-C), but did not improve the BMI compared with the control groups [102].

##### Effects of Tai Chi on Diabetes

According to one systematic review, which meta-analyzed 17 trials regarding the use of tai chi in patients with T2D, tai chi was noted to significantly reduce the FBG, HbA1c, TC, triglyceride (TG), and BMI, but not LDL-C and HDL-C, compared with the control group [90]. According to another meta-analysis study that examined the effectiveness of tai chi in patients with T2D (meta-analysis of 14 trials), tai chi was noted to significantly reduce the FBG, HbA1c, and PPBG compared with the non-exercise control groups [91]. The common observation in both meta-analyses was that tai chi decrease FBG and HbA1c in patients with T2D.

##### Effects of Qigong on Diabetes

According to a meta-analysis of 21 randomized controlled trials (RCTs) that examined the effects of qigong in adults with T2D, qigong significantly reduced FBG, HbA1c, and PPBG [89]. According to another meta-analysis study (11 RCTs) regarding qigong’s effectiveness in adults with T2D, qigong significantly reduced FBG, PPBG, HbA1c, TG, and HDL-C, but no significant changes were noted related to TC and LDL-C [103]. Both meta-analyses had a common observation of qigong decreasing the FBG, HbA1c, and PPBG in patients with T2D.

##### Effects of Yoga on Diabetes

According to a meta-analysis of 12 RCTs that examined the effects of yoga in adults with T2D, yoga significantly reduced the FBG, HbA1c, PPBG, TC, HDL-C, and LDL-C, but did not significantly reduce the TG [94]. Another meta-analysis study that examined the effectiveness of yoga in adults with T2D (meta-analysis of 23 studies) determined that yoga improved HbA1c, FBG, and PPBG compared with the control groups. Moreover, yoga significantly improved other risk factors, such as lipid profile, blood pressure, BMI, waist–hip ratio, and cortisol level [93]. In a meta-analysis using 17 RCTs regarding the effects of yoga in adults with T2D, yoga improved HbA1c, FBG, and PPBG compared with control groups [95]. Therefore, all the three meta-analyses, which investigated the effects of yoga in patients with T2D, reported that yoga reduces FBG, HbA1c, and PPBG in patients with T2D. A meta-analysis comprising 42 RCTs examined the effectiveness of yoga (w/wo MBSR) against active controls in all populations, and reported a significant reduction in FBG, TC, and LDL-C, with unchanged TG and HDL-C [92]. Regardless of the group studied, a decrease in blood glucose was consistently observed in the yoga group.

#### 2.1.2. Effects of Combined Practices (Sitting Meditation and Moving Meditation) on Diabetes

##### Mindfulness-Based Intervention

Meta-analysis of eight RCTs conducted on diabetics (including types 1 and 2) revealed that MBI has a beneficial effect on HbA1c, diabetes-related distress, depression, and stress [87]. The fact that MBI positively affects HbA1c is consistent with previous meta-analyses [104,105]. Another study that conducted a meta-analysis of nine RCTs involving diabetics (including types 1 and 2) observed that MBSR and mindful cognitive therapy (MBCT) improved depression, the mental health composite score of quality of life (QOL), and HbA1c [87]. Sensitivity analysis revealed that the positive effect on HbA1c disappeared when long-term tracking studies (more than 6 months) were excluded, suggesting that the effects of MBSR or MBCT on HbA1c takes time to happen.

##### Buddhist Walking Meditation

According to an RCT study that compared the effects of Buddhist walking meditation to traditional walking in adults with T2D, Buddhist walking meditation was noted to significantly reduce the HbA1c, but did not significantly alter the FBG, TC, HDL-C, LDL-C, and TG [106].

##### Brain Education Meditation

According to the RCT study that compared the effects of BEM (a mixed method which combines static and moving meditations) in patients with T2D or high blood pressure with that of the effects in the health education group, BEM significantly reduced LDL-C [107]. In addition, a cross-sectional study that compared long-term women meditators and women non-meditators revealed a significantly higher blood glucose levels in the postmenopausal participants than the premenopausal participants in the control group, whereas no such increase was observed in the BEM group [108]. 

#### 2.1.3. The Effects of Other Practices on Diabetes-Related Factors

In an imaginal retraining RCT concerning the reduction of craving for high-calorie food in 384 overweight and obese women, a 6-week imaginal retraining without diet or lifestyle recommendation significantly reduced body weight compared with the waitlist control [109]. In an RCT study that examined the effects of 12 weeks of pilates on glycemic control of older women with T2D, pilates was noted to significantly reduce PPBG and HbA1c [110].

### 2.2. Potential Mechanism for Diabetes-Related Effects of Mind–Body Intervention

In studies using rodents, parental stress, fetal stress, and post-birth adversities were observed to affect the epigenetic modifications in the promoter of the glucocorticoid receptor. When an individual exposed to stress becomes an adult, the resulting epigenetic changes might affect the coping behavior in adverse conditions and this behavior pattern might transmit transgenerationally. However, these transgenerational epigenetic marks can be reversed through environmental abundance, including favorable experiences, thereby suggesting that environmental abundance can be a powerful intervention in reversing epigenetic programming [111,112]. The environmental abundance used in the above study is cognitive and somatosensory stimulation, exercise, and a visual stimulation-rich environment. In humans, MBI makes one aware of the current moment and one’s body condition through breathing and improves the connection between the body and the brain through soft and slow motion. Therefore, MBI provides a component corresponding to the environmental abundance. Indeed, MBI changes epigenetic modifications, as well as mental and physical functions as follows [113,114] (Table 2).

#### 2.2.1. Changes in Epigenetic Modifications Related to Glucose/Lipid Metabolism and Inflammation through Mind–Body Intervention

MBI-induced epigenetic changes reported so far include DNA methylation [114,115,116,117,118,119,120,121] and histone modification [115], but there are no reports regarding non-coding RNA at present (Table 2). As mentioned earlier, accumulated research has revealed that MBI positively improves the blood glucose and lipids in people with diabetes. To investigate how mindfulness affects the epigenetic pathways, García–Campayo et al. [117] compared the methylation profiles obtained from the circulating lymphocytes of non-meditators and experienced meditators with more than 10 years of experience. They identified 64 differentially methylated regions and found that the 43 genes contained in them were related to glucose homeostasis, lipid metabolism, protein folding, neurotransmission, and inflammatory pathway regulation [117]. Most of these genes were associated with neurologic disorders, psychiatric illnesses, cardiovascular diseases, and cancer. Furthermore, in silico analysis predicted that epigenetic reactions to the mindfulness practice regulate inflammatory pathways dependent on the tumor necrosis factor (TNF) alpha and nuclear factor kappa light chain enhancer of activated B cells (NF-kB) signaling.

The authors performed GO enrichment analysis to characterize the functions of genes that have more mindfulness-related differentially methylated regions (DMRs). In the cellular component category, several GO terms were related to different lipoprotein particles, whereas the most strongly related GO term was phospholipase binding in molecular function category. Among differentially methylated genes, several genes functioning in lipid metabolism or related functions (e.g., *APOB*, *APOC2*, *HRH1*, *PTCH1*, *CLEC11A*, *NCOR*) were included. In differential genes, the most frequently presented top canonical pathways were LXR/RXR and FXR/RXR, which are essential pathways in regulating the atherosclerosis signaling pathway, as well as cholesterol, fatty acid, and glucose homeostasis. Moreover, DMRs enriched in transcription factor-binding motifs, and Meis 3 or Mak that are transcription factors related to pancreatic beta cell survival or insulin metabolism were also included. In response to oxidative stress, transcription factors of several motifs act commonly into directing the upregulation of Nrf2 which exerts anti-inflammatory and neuroprotective functions. When predicting and analyzing the upstream regulator of the 43 differentially methylated genes by meditation, the cytokine TNF was noted to have the highest correlation. TNF is a cytokine involved in a wide range of human diseases, and previous studies have also revealed associations between meditation and TNF [122,123].

In García’s study [117], which analyzed peripheral blood mononuclear cell DNA methylation compared with meditation-naïve controls, meditators contained changed epigenetic marks associated with glucose and lipid metabolism as well as inflammation, suggesting related functional improvements through MBI, supporting the possibility of using MBIs to improve glucose and lipid metabolism, as well as inflammatory function. In a study which analyzed the same DNA samples, the *SERPINB9* gene, which is differentially methylated by meditation [118], has been known to be associated with inflammation and insulin resistance in coronary atherosclerosis [124]. The methods used in this study are cross-sectional studies, thus the causal relationship is unknown. Therefore, it is necessary to examine epigenetic changes caused by meditation with the research design of RCTs in the future to reveal the causal relationship. The meditation method used by García et al. [117] is a mindfulness meditation. Hence, it would be noteworthy to examine if other meditation techniques, such as moving meditation, can induce different epigenetic modifications.

#### 2.2.2. Reduction of Psychological Stress, A Risk Factor of Type 2 Diabetes, through Mind–Body Intervention

MBI has been shown to be effective in reducing negative psychological factors, including depression. A meta-analysis of 38 RCTs that examined the effect of meditation and MBI on healthcare professionals revealed that the intervention significantly reduced anxiety, depression, psychological distress, stress, and improved overall well-being [125]. A meta-analysis of 6 clinical studies involving 405 pregnant women revealed that yoga-based interventions significantly decreased depression during pregnancy [126]. Furthermore, a meta-analysis that studied the effects of qigong and tai chi on cancer survivors revealed that the intervention was significantly effective on fatigue symptoms (7 studies), sleep quality (2 studies), and positive trends, but not statistically significantly effective on anxiety (3 studies), stress (2 studies), depressive symptoms (4 studies), or QOL (5 studies) [127].

Therefore, we explored whether epigenetic changes occur when MBI exerts a positive effect on psychological factors. Bishop et al. [119] performed a study that could provide an answer to this. They conducted an MBSR on patients with post-traumatic stress disorder (PTSD) to investigate the differences in DNA methylation in the peripheral blood samples between responders and non-responders to the MBSR intervention. They observed that methylation in CpG within the FKBP5 gene region containing the glucocorticoid response element was decreased in responders and increased in non-responders, thereby suggesting that effective meditation is associated with stress-related pathways at the molecular level [119].

Accumulated brain imaging studies support the reduction of stress, depression, anxiety, and PTSD through MBI. A meta-analysis of 21 neuroimaging studies (300 meditation practitioners) revealed that 8 brain regions were consistently altered in meditators regardless of the meditation method. Among these regions, the orbitofrontal cortex and anterior and mid cingulate were specifically associated with self and emotion regulation [128]. Therefore, MBI changes the brain structures and allows heightened self-monitoring and a better emotional regulation. These structural changes of the brain caused by MBI explain how MBI brings apparent beneficial effects on depression, anxiety, and stress.

Notably, psychological stress is a predictor for the onset of T2D and a prognostic factor for existing T2D [28]. Because of the proven effects of MBI in reducing psychological stress, it might also help in reducing the T2D risk induced through psychological stress. Because stress changes the neuroendocrine (cortisol), inflammatory, and autonomic neural pathways [28], it is of interest to ascertain how MBI, which effectively relieves stress and controls blood glucose, alters each of these pathways.

##### Cortisol Secretion and Glycemic Control through Mind–Body Intervention

Cortisol affects glucose homeostasis [29]. Its circulation induces the release of glucose and lipids [28]. Notably, evening cortisol was increased in patients with diabetes. MBI might alter the HPA axis, thereby controlling the blood glucose through cortisol secretory regulation, which is an output of the HPA axis. According to a meta-analysis of 42 RCTs, which investigated the effects of yoga asanas with or without MBSR on stress-related physiological measures in all populations, yoga practice seemed to reduce waking, as well as afternoon and evening salivary cortisol [92]. Moreover, a meta-analysis of 23 trials that studied the effects of yoga in patients with T2D revealed that yoga significantly reduced the afternoon, evening, and waking cortisol levels, but did not reduce the 30 or 60 min post-waking and mid-morning cortisol levels, or the cortisol slope [93]. According to a meta-analysis related to qigong, the cortisol level was not significantly changed [129]. Therefore, the blood glucose level altered through MBI might be partially contributed to by the cortisol-mediated pathway.

##### Autonomic Nervous System Changes and Glycemic Control through Mind–Body Intervention

Increased blood pressure is a well-known risk factor for diabetes. A meta-analysis of prospective studies revealed that an increase in blood pressure correlated with an increase in the risk of diabetes [48]. Studies have revealed MBI to be effective in reducing blood pressure. In a meta-analysis of 9 trials investigating the effects of TM on blood pressure in adults with hypertension or cardiovascular disease, the intragroup analysis revealed that systolic and diastolic blood pressures were significantly reduced through the intervention [130]. In a meta-analysis that analyzed 49 studies on the effects of yoga in middle-aged overweight adults with high blood pressure, yoga significantly reduced both the systolic and diastolic blood pressures compared with the controls [131]. Another meta-analysis of 13 studies on meditation and yoga revealed that these interventions reduced both systolic and diastolic blood pressures [132]. Therefore, decreasing blood pressure through MBI seemed to partially contribute to reducing the risk of diabetes.

##### Inflammation Reduction and Glycemic Control through Mind–Body Intervention

Inflammation is a factor that increases the risk of T2D [52,54]. Several studies have reported a reduction in inflammatory markers through MBI. According to a study that systematically reviewed 20 RCTs on mindfulness meditation, NF-kB transcription activity and CRP level were reduced in mindfulness meditation practitioners compared to the general public, suggesting that inflammation was decreased [73]. A single intensive mindfulness meditation of 8 h significantly reduced the expression of histone deacetylase genes (*HDAC2*,*3*,*9*), altered the global modification of histones (H4ac; H3K4 me3), and decreased the expression of pro-inflammatory genes (*RIPK2*, *COX2*) in peripheral blood samples of meditation experts compared with those of the meditation-novices who joined a leisure activity of 8 h [115], thereby indicating that an MBI-induced reduction of pro-inflammatory gene expression occurs along with epigenetic alterations within a day in MBI experts. In a meta-analysis of 34 RCT studies (2219 participants), which investigated the immune outcome measures changed through MBI (tai chi, qigong, meditation, yoga), the CRP level was significantly reduced through MBI, whereas IL-6 and TNF-alpha levels were not significantly altered [133]. In addition, it has been reported that yoga and mindfulness practice reduce the expression of pro-inflammatory genes in the blood cells [134,135]. In a study comparing a yoga-performing group with a control group among women reporting psychological distress, the yoga group showed a lower level of methylation in the *TNF* gene associated with inflammation than in the control group in peripheral blood samples [120]. Notably, the decrease in methylation of the *TNF-alpha* gene promoter in blood mononuclear cell DNA is associated with weight loss in obese men, as well as with the reduction of circulating levels of baseline TNF-alpha [136]. Because inflammation is a factor that increases the risk of T2D, a decrease in the expression of pro-inflammatory factors through MBI could decrease inflammation, thereby decreasing the risk of T2D.

#### 2.2.3. Delayed Epigenetic Age through Mind–Body Intervention and Its Relation to Type 2 Diabetes

Recent studies have revealed that biological aging measurements are possible by analyzing the methylation of CpG sites in the genome [137,138]. Deterioration of important genome maintenance mechanisms might occur due to aging, resulting in changes in DNA methylation over time. The results of research in this field thus far suggest that MBI might potentially delay or reverse aging-related changes in the epigenome. Chaixs and colleagues [116] used Horvath’s calculator [137], which calculates the biological aging rate by measuring DNA methylation, to compare the aging rate between experienced meditators (18 participants) and meditation-naïve individuals (20 participants) using a cross-sectional design in peripheral blood mononuclear cell samples. It was observed that the aging rate was significantly higher in people above 52 years of age than those below 52 years of age in the control group. However, in experienced meditators, this epigenetic aging difference was not observed between two different chronological age groups. In addition, the epigenetic aging rate in meditators was significantly reduced proportionally to the number of years of meditation. This finding suggested that incorporating meditation into daily routines might slow the epigenetic clock, giving potential health benefits in the long run [116]. After this study, it was revealed that short meditation interventions (8 h) performed by experienced meditators could quickly affect the methylome of genes related to immune metabolism, inflammation, and aging [114].

A recently reported study by Mendioroz et al. [118] investigated 14 differentially methylated regions in peripheral blood samples, present in the subtelomeric region, which were identified in long-term meditators compared with the controls in their previous work [117]. The telomere length of long-term meditators positively correlated with the methylation level of the *GPR31* gene but correlated inversely to the methylation level of the *SERPINB9* gene. In addition, the correlation between telomere length and age that was observed in the general population was no longer found in long-term meditators. Hence, these results suggest that long-term meditation might be associated with epigenetic mechanisms related to certain gene-specific DNA methylation changes in distinct subtelomeric regions. Moreover, delays in epigenetic aging rates were demonstrated in the analysis of epigenetic effects of tai chi, a moving meditation [121]. Approximately 66 methylation sites of experienced tai chi performers and the general population were compared using their saliva sample, and a significant difference was found in 6 CpG sites of 3 different chromosomes. Methylation changes in this area relative to age were significantly slower in the tai chi cohort compared to that of the control cohort [121].

Because this research field is relatively new, several studies have been performed using a cross-sectional design or with a small number of people; therefore, more research should be performed to prove the causal relationships between MBI and DNA methylation. It is generally well-known that fasting glucose levels increase as age increases [139,140,141]. Therefore, the delay of aging rates by epigenetic marks of aging-related genes through MBI [114,116,121] might partly contribute to the effect of MBI on reduction of blood glucose (Table 1).

#### 2.2.4. Glycemic Control through Lifestyle Changes with Mind–Body Intervention

People with high stress have unhealthier behaviors in smoking, exercising, alcohol drinking, and weight management compared with those with low stress [142]. If the patient with diabetes had depression, the non-adherence to a healthy behavior increased [31]. Therefore, stress and depression reduction through MBI [143,144,145] might affect behavior, which may help to lower T2D risks induced through unhealthy behaviors. Notably, it has been confirmed that adhering to an optimal behavior is effective in reaching the targeted HbA1c [146,147].

### 2.3. Epigenetic Changes Induced by Non-Pharmacological Interventions in Addition to Mind–Body Interventions and Their Effects on Diabetes

In addition to MBI, interventions with the aim of improving living environments and behavior (e.g., education, exercise, diet, sleep) were associated with changes in DNA methylation profiles [148,149]. Furthermore, elements of MBI have been combined with existing cognitive and psychological interventions [150,151]. Changes were observed in the DNA methylation profiles in response to cognitive behavioral therapy and social support [152,153]. A meta-analysis that used 8 RCT trials comparing cognitive behavior therapy (CBT) to non-CBT in patients with diabetes (including types 1 and 2) revealed that CBT significantly reduced HbA1c compared with the control (non-CBT) [97]. In another meta-analysis of 10 RCTs comparing CBT or CBT-based therapy with non-CBT in patients with diabetes with depression (including types 1 and 2), the interventions significantly reduced FBG in the CBT group (or CBT-based therapy) compared with non-CBT. However, no significant differences were noted related to HbA1c between the groups [96]. In a meta-analysis regarding the effects of psychoeducational intervention on glycemic control and diabetes-specific emotional distress (DSD), both HbA1c (23 RCTs) and DSD (32 RCTs) were significantly reduced through the intervention [154]. Psychotherapies also slightly improved the HbA1c of diabetics [104,105]. 

Research on exercise has examined whether short-term or long-term exercise affects DNA methylation in skeletal muscle and fat tissues [3,155,156]. Studies of exercise in patients with diabetes have reported differences in the effectiveness depending on the intensity of exercise. A meta-analysis of 10 RCTs using high-intensity interval training (HIIT) for T2D revealed that HIIT significantly reduced HbA1c compared with non-exercise or low-intensity training and was not different from moderate-intensity training [100]. In a meta-analysis using 8 randomized trials that compared higher and lower intensity training in patients with T2D, higher intensity training exhibited a significant reduction in HbA1c, but no differences were noted regarding FBG compared with lower intensity training [98]. A meta-analysis of 9 trials that compared the effects of exercise and non-exercise in patients with T2D revealed that the exercise group had a significant decrease in HbA1c than the non-exercise group [99]. The high intensity of exercise effectively reduced the blood glucose, and MBI might be considered a relatively mild intensity exercise. However, MBI is likely to help improve cardiovascular function [157,158], potentially due to its large focus on breathing control, and might help with blood glucose management in a different way than exercise by modulating the stress axis and properly activating the parasympathetic system in daily life. 

**Figure 1:**
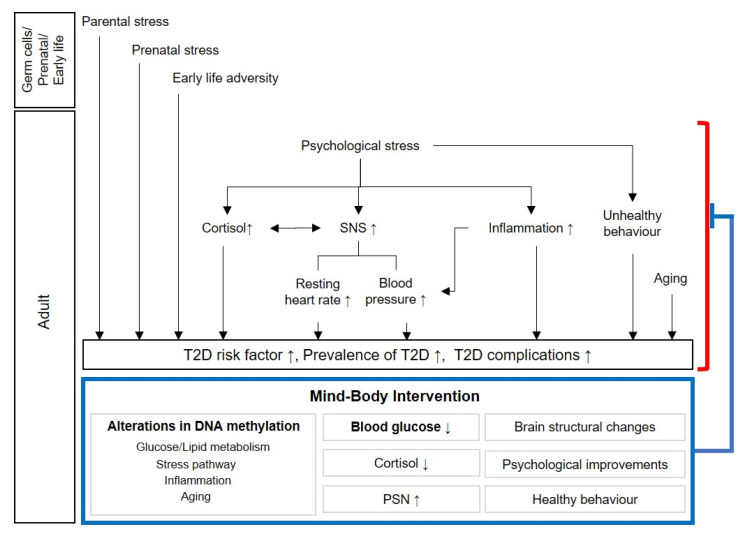
Potential causes contributing to type 2 diabetes (red) and mind–body intervention-induced beneficial changes including altered epigenetic modification (blue). Abbreviations: SNS, sympathetic nervous system; T2D, type 2 diabetes; PSN, parasympathetic nervous system; ↑, increased; ↓, reduced.

MBI improves health potentially through managing both mind and body to induce relaxation and cultivate a sense of acceptance, thereby altering the stress response (Figure 1). Exercise improves health through energy consumption, and CBT or lifestyle education through changing perception. These different approaches have been reported to have significant effects on diabetes management and prevention, respectively. Therefore, a possible method can be selected according to the individual’s situation for the long-term blood glucose management in patients with diabetes. 

## 3. Conclusions

T2D is a chronic condition necessitating the use of lifetime medications, with potential side effects if the medication is less specific. Therefore, because several MBIs can significantly contribute to blood glucose control, long-term diabetic care can incorporate MBI as a complementary method. Notably, an effective individualized MBI protocol organized based on glycemic control evidence might be highly beneficial in individuals with diabetes or prediabetes. Regarding the effects of MBI on glycemic control, this current review mainly focused on individuals with diabetes but not the prediabetic population. Long-term follow-up investigations that explores whether MBI prevents T2D in individuals with disease risks will provide a better understanding of diabetes prevention through MBI. Furthermore, because the epigenetic modifications are reversible, studies and clinical guidelines should explore how long the glucose metabolism-related positive changes induced by MBIs are maintained and determine how often these interventions need to be performed to obtain lifetime effects. These findings will guide the actual glycemic management in patients with T2D as well as the general population.

Exercise can reprogram the sperm methylome in humans [159]. Exercise and MBI share some benefits in common. However, MBI is milder in intensity and stronger in mental training than regular exercise. Therefore, questions like whether MBI can change the epigenetic marks in human reproductive cells and whether these changes are inherited remain to be answered. Even if the same exercise is performed, differences in skill of execution in exercise seem to induce different epigenetic and transcriptional responses [155]. Thus, the MBI studies should also be designed with care as the proficiency of the subjects may affect the results. In addition, cross-sectional studies have the disadvantage of not being able to distinguish whether epigenetic changes have caused participants to perform MBI or whether these epigenetic changes were induced by MBI. Research on epigenetic changes in the field of MBI is still relatively new, and therefore more longitudinal studies and sample numbers are required in the future to further explore all aspects accurately.

Although there are only few epigenetic studies on MBI and without enough longitudinal studies on this topic, based on the research so far, the rate and level of DNA methylation modification seem to be affected by MBI, potentially leading to less modification [117,119,120]. Notably, in this less methylated state, the machinery for gene expression may be able to access the gene more easily. It is intriguing to hypothesize that this may increase the plasticity of gene expression, providing a molecular environment for a more flexible response to changing environments.

## Figures and Tables

**Table 1:** Recent meta-analyses about the effects of mind–body intervention and other protocols on regulation of blood glucose and lipid profiles.

Meta-Analysis	Study Type	Participants		Intervention	Duration, Frequency, Intervention Length	Control	Number of Trials (Number of Participants)	Outcomes (Compared to the Control)
Ni et al., 2020 (Journal of Nursing Scholarship) [87]	RCT	Patients with types 1 and type 2 diabetes	Mind–body intervention	Mindfulness-based intervention (MBSR, MBCT)	90–150 min/session, 1 session/week, 8–9 weeks	Usual care, waitlist, health education without any mindful component	6 studies (*n* = 578)	↓HbA1c
Ni et al., 2020 (J Diabetes Investing) [88]	RCT	Patients with types 1 and type 2 diabetes		Mindfulness-based intervention (MBSR, mindful eating intervention, MBCT, MBCT + MBSR)	30–150 min/session, 1–7 sessions/week, 8–12 weeks	Usual care, Smart choices DSME-based intervention, waitlist, CBT	7 studies (*n* = 665)	↓HbA1c
Meng et al., 2018 [89]	RCT	Patients with type 2 diabetes		Qigong	NR, 20–90 min/session, 1–5 sessions/week, 2–12 months	No exercise	17 studies (*n* = 966)	↓FBG
						Other aerobic exercises	5 studies (*n* = 389)	= FBG (*p* = 0.07)
						Antiresistance exercise	1 study (*n* = 22)	↓FBG
						No exercise	16 studies (*n* = 834)	↓HbA1c
						Other aerobic exercises	5 studies (*n* = 389)	↓HbA1c
						Antiresistance exercise	1 study (*n* = 22)	= HbA1c
						No exercise	6 studies (*n* = 330)	↓PPBG
						Other aerobic exercises	1 study (*n* = 122)	↓PPBG
Xia et al., 2019 [90]	RCT	Patients with type 2 diabetes		Tai chi	30–120 min/session, 2–14 sessions/week, 2–6 months	Usual care, standard treatment, any kind of exercise	13 studies (*n* = 616)	↓FBG
							9 studies (*n* = 517)	↓HbA1c
							7 studies (*n* = 343)	↓TC
							8 studies (*n* = 359)	↓TG
							6 studies (*n* = 290)	= HDL-C
							6 studies (*n* = 290)	= LDL-C
							6 studies (*n* = 296)	↓BMI
Chao et al., 2018 [91]	RCT	Patients with type 2 diabetes		Tai chi	15–60 min/session, 2–7 sessions/week, 1–6 months	Non-exercise	10 studies (*n* = 489)	↓FBG
						Other aerobic exercise	7 studies (*n* = 342)	= FBG
						Non-exercise	7 studies (*n* = 293)	↓HbA1c
						Other aerobic exercise	7 studies (*n* = 372)	= HbA1c
						Non-exercise	5 studies (*n* = 162)	↓PPBG
						Other aerobic exercise	3 studies (*n* = 84)	= PPBG
Xia et al., 2020 [87]	RCT	Patients with type 2 diabetes		Meditative movements (tai chi or qigong or yoga)	NR, 10–120 min/session, 2–7 sessions/week, 6–36 weeks	Any type of control group	19 studies (*n* = 1505)	↓FBG
							15 studies (*n* = 1116)	↓HbA1c
							5 studies (*n* = 624)	↓PPBG
							12 studies (*n* = 1110)	↓TC
							8 studies (*n* = 844)	↓LDL-C
							10 studies (*n* = 991)	↓TG
							9 studies (*n* = 938)	↑HDL-C
							11 studies (*n* = 915)	=BMI
Pascoe et al., 2017 [92]	RCT	All population		Yoga *w*/*wo* MBSR	45–120 min/sessions, 1–7 sessions/week, 2–14 months	Active controls (exercise, physical activity, health education, social support, stretching, progressive muscle relaxation, other counselling/therapy)	7 studies (*n* = 534)	↓FBG
							6 studies (*n* = 389)	↓TC
							6 studies (*n* = 389)	↓LDL-C
							7 studies (*n* = 560)	= TG
							7 studies (*n* = 560)	= HDL-C
Thind et al., 2017 [93] *	RCT, non-RCT	Patients with type 2 diabetes		Yoga (not specified, hatha, Sudarshan kriya)	50–240 min/session, total 12~182 h (<1–26 weeks)	Usual care, waitlist, exercise only, exercise plus lifestyle education	18 studies (*n* = 2212)	↓HbA1c
							21 studies (*n* = 2081)	↓FBG
							14 studies (*n* = 1473)	↓PPBG
							16 studies (*n* = 1880)	↑HDL-C
							16 studies (*n* = 1838)	↓LDL-C
							16 studies (*n* = 1895)	↓TC
							14 studies (*n* = 1790)	↓TG
							9 studies (*n* = 1260)	↓BMI
Cui et al., 2016 [94]	RCT	Patients with type 2 diabetes		Yoga (hatha, asana, pranaya, Sudarshan kriya, shavasana)	30–120 min/session, 1–7 sessions/week, 15 days–9 months	Usual care, physical exercises, life style education, brisk walking, waitlist	9 studies (*n* = 805)	↓FBG
							7 studies (*n* = 718)	↓HbA1c
							4 studies (*n* = 527)	↓PPBG
							5 studies (*n* = 618)	↓TC
							4 studies (*n* = 588)	↑HDL-C
							5 studies (*n* = 618)	↓LDL-C
							4 studies (*n* = 588)	=TG
Kumar et al., 2016 [95]	RCT	Patients with type 2 diabetes		Yoga (asana, pranayama, hatha, relaxation, diaphragmatic breathing in supine position)	30–120 min/session, 1–7 sessions/week, 40 days–6 months	Usual care, walking, education	17 studies (*n* = 1358)	↓FBG
							9 studies (*n* = 659)	↓PPBG
							13 studies (*n* = 1097)	↓HbA1c
Li et al., 2017 [96]	RCT	Diabetes patients (type1 or 2) with clinically relevant depression	CBT	CBT or a therapy based on CBT	NR, 45–90 min/sessions, 1–2 sessions/week, 2–12 months	Usual care, diabetes education, usual care, waitlist	7 studies (*n* = 759)	= HbA1c
							5 studies (*n* = 303) short-term effect	= HbA1c
							6 studies (*n* = 705) long-term effect	= HbA1c
							3 studies (*n* = 175)	↓FBG
Uchendu et al., 2017 [97]	RCT	Patients with Type 1 or Type 2 diabetes		CBT	30–120 min/sessions, 1–2 sessions/week, 6–16 weeks	Non-CBT	16 studies (*n* = 1375)	↓HbA1c
Liubaoerjijin et al., 2016 [98]	RCT	Patients with type 2 diabetes	Exercise	Higher intensity training (walking/cycling/running/treadmill/XC ski)	NR, 15–60 min/session, 3–6 sessions/week, 12–25 weeks	Lower intensity training (Walking/Cycling/Treadmill/XC ski)	8 studies (*n* = 233)	↓HbA1c
								= FBG
Boule et al., 2001 [99]	RCT, CCT	Patients with type 2 diabetes		Exercise	40–90 min/sessions, 2–6 sessions/week, 8–22 weeks	Non-exercise	11 studies (*n* = 310)	↓HbA1c
				Exercise with diet	30–45 min/session, 3–3.5 sessions/week, 13–52 weeks	Non-exercise, Non-diet	3 studies (*n* = 142)	↓HbA1c
Lora–Pozo et al., 2019 [100]	RCT	Patients with type 2 diabetes		High-intensity interval training (64–90% VO2max or 77–95% heart rate max)	21–60 min/session, 3–5 sessions/week, 12–16 weeks	Non-exercise	2 studies (*n* = 43)	↓HbA1c
					30–60 min/session, 2–5 sessions/week, 12–16 weeks	Moderate-intensity training	4 studies (*n* = 105)	= HbA1c
					60–83 min/session, 2–5 sessions/week, 16–48 weeks	Low-intensity training	2 studies (*n* = 312)	↓HbA1c

Abbreviations: MBSR, mindfulness-based stress reduction; MBCT, mindfulness-based cognitive therapy; DSME, diabetes self-management education; CBT, cognitive behavior therapy; HbA1c, hemoglobin A1c; FBG, fasting blood glucose; PPBG, post-prandial blood glucose; TC, total cholesterol; LDL-C, low-density lipoprotein cholesterol; HDL-C, high-density lipoprotein cholesterol; TG, triglyceride; BMI, body mass index; NR, not reported; RCT, randomized controlled trial; CCT, nonrandomized controlled trial; ↑, increased; ↓, reduced; =, no difference. * The numbers of participants used in meta-analysis were not separately described in Thind et al., (2017). The above-indicated numbers are calculated according to the original references provided in Table 1 of Thind et al., (2017).

**Table 2:** Changes in epigenetic marks related to mind–body intervention.

Ref	Study Type	Intervention			Control			Sample	Changes in Epigenetic Marks	Differentially Methylated Area	Related Functions
		Participants	Protocol	Duration	Participants	Protocol	Duration				
Kaliman et al., 2014 [115]	L	Experienced meditators (*n* = 19) (a daily meditation practice spanning a minimum of 3 years, ≥30 min/day, ≥3 intensive retreats lasting 5 or more days)	Intensive meditation practice (a day-long session of the MBSR, which is routinely used in North-American hospitals)	8 h	People with no meditation experience (*n* = 21)	Leisure activity (reading, watching documentaries or playing computer games, and walking)	8 h	PBMC	↑Global acetylation of histone H4 (H4ac) ↓Trimethylation of histone H3 lysine 4 (H3K4me3)		
Chaix et al., 2020 [114]		Experienced meditators (*n* = 17) (Same participant pools of Kaliman et al., 2014)			People with no meditation experience (*n* = 17) (Same participant pools of Kaliman et al., 2014.)				No significant baseline differences in methylation profiles between groups 61 DMRs after the intervention in the meditation group compared to the control group • DMRs include genes related with immune response, inflammation, ageing	*ACADM*, *CPT1A*, *HSD17B4*	Fatty acid metabolism
										*SAP18*, *EIF1B*, *NCBP2*	RNA transport
										*APITD1*, *ERCC1*	DNA repair
										*KLF15*	Glucose homeostasis, stress response, inflammation
										*EGR1*	DNA damage, immunity, inflammatory responses
										*SP3*	DNA damage, immunity, hematopoiesis, expression regulation of anti-inflammatory molecules such as IL-10 and COX-2
										*SP4*	Inflammatory and neuropathic persistent pain states, dendrite patterning, neurotransmission
										*EGR2*	Involved in immunity and inflammatory processes, as well as various neuropathies
Chaix et al., 2017 [116]	C	Experienced meditators (*n* = 17) (Same participant pools of Kaliman et al., 2014)	-	-	People with no meditation experience (*n* = 17) (Same participant pools of Kaliman et al., 2014)	-	-	PBMC	Epigenetic age in controls: Older (age ≥ 52) > younger (age < 52) • Epigenetic age in meditators: Older (age ≥ 52) = younger (age < 52)	-	-
García–Campayo et al., 2018 [117]	C	Experienced mindfulness meditators (*n* = 17) (≥ 10 years in total, ≥ 60 min/day)	-	-	Healthy relatives and friends of the meditators who had a similar lifestyle (*n* = 17)	-	-	PBMC	64 DMRs corresponding to 43 genes ↓Methylation in 70.3% of mindfulness-related DMRs Almost half of the DMRs involved genes linked to common human diseases, such as cardiovascular diseases 23.4% of DMRs located at subtelomeric regions Lipid metabolism and atherosclerosis signaling pathway: Significantly enriched in mindfulness-related DMRs • TNF, NF-kB signaling: Crucial regulators of the mindfulness-related genes	*Meis3, Mafk*	Glucose homeostasis
										*APOB*, *APOC2*, *HRH1*, *PTCH1*, *CLEC11A*, *NCOR*	Lipid metabolism
										*TNFα*, *NF-kB*, *Nrf2*	Inflammation
Mendioroz et al., 2020 [118]	C	Experienced meditators (*n* = 17) (Same participant pools of García–Campayo et al., 2017)	-	-	Healthy relatives and friends of the meditators who had a similar lifestyle (*n* = 17) (Same participant pools of García–Campayo et al., 2017)	-	-	PBMC	Positive correlation between methylation level of *GPR31* and telomere length in meditator Negative correlation between methylation level of *SERPINB9* and telomere length in meditator Negative correlation between methylation level of the intergenic CpG island within the subtelomeric region of chromosome 4 short arm and telomere length in meditator No correlation between age and telomere length in meditators	*GPR31*	Tumorigenesis, extravasation, and metastasis
										*SERPINB9*	Inhibition of apoptosis, inflammation, insulin resistance in coronary atherosclerosis
										Intergenic CpG island within the subtelomeric region of chromosome 4 short arm	-
Bishop et al., 2018 [119]	L	PTSD patients of MBSR responder (*n* = 11)	MBSR	9 weeks	PTSD patients of MBSR non-responder (*n* = 11)	MBSR	9 weeks	PBMC	↓Methylation in responders	*FKBP5*	Stress-related pathway (glucocorticoid receptor regulation)
									↑Methylation in non-responders		
Harkess et al., 2016 [120]	L,C	Women reporting psychological distress (≥ 16 on Kessler Psychological Distress Scale) (*n* = 15)	Yoga	8 weeks	Women reporting psychological distress (≥ 26 on Kessler Psychological Distress Scale) (*n* = 11)	Control (waitlist)	8 weeks	PBMC	↓Methylation (cross-sectional: Post-intervention)	*TNF*	Inflammation
Ren et al., 2012 [121]	C	Women tai chi practitioners (*n* = 237) (≥ 3 years)	-	-	Women with no practice of tai chi (*n* = 263)	-	-	Saliva	Significantly slow age-related methylation dynamics in tai chi group compared to the control group in six age-related CpG marks	Age-related CpGs (*Rad50_2*, *17P_7*, *G6PD_6*, *G6PD_7*, *Rad50_10*, *Xp13_1*)	Aging

Abbreviations: L, longitudinal; C, cross-sectional; PBMC, peripheral blood mononuclear cells, DMR, differentially methylated regions; PTSD, posttraumatic stress disorder; MBSR, mindfulness-based stress reduction; ↑, increased; ↓, reduced.

## Data Availability

Data are contained within the article.
